# Defining and targeting patterns of T cell dysfunction in inborn errors of immunity

**DOI:** 10.3389/fimmu.2022.932715

**Published:** 2022-09-14

**Authors:** Jose S. Campos, Sarah E. Henrickson

**Affiliations:** ^1^ Division of Allergy and Immunology, Department of Pediatrics, Children’s Hospital of Philadelphia, Philadelphia, PA, United States; ^2^ Institute for Immunology, University of Pennsylvania Perelman School of Medicine, Philadelphia, PA, United States; ^3^ Department of Microbiology, University of Pennsylvania Perelman School of Medicine, Philadelphia, PA, United States

**Keywords:** inborn error of immunity (IEI), human immunology, CRISPR (clustered regularly interspaced short palindromic repeat)/Cas9 (CRISPR associated protein 9)-mediated genome editing, t cell exhaustion, precision medicine

## Abstract

Inborn errors of immunity (IEIs) are a group of more than 450 monogenic disorders that impair immune development and function. A subset of IEIs blend increased susceptibility to infection, autoimmunity, and malignancy and are known collectively as primary immune regulatory disorders (PIRDs). While many aspects of immune function are altered in PIRDs, one key impact is on T-cell function. By their nature, PIRDs provide unique insights into human T-cell signaling; alterations in individual signaling molecules tune downstream signaling pathways and effector function. Quantifying T-cell dysfunction in PIRDs and the underlying causative mechanisms is critical to identifying existing therapies and potential novel therapeutic targets to treat our rare patients and gain deeper insight into the basic mechanisms of T-cell function. Though there are many types of T-cell dysfunction, here we will focus on T-cell exhaustion, a key pathophysiological state. Exhaustion has been described in both human and mouse models of disease, where the chronic presence of antigen and inflammation (e.g., chronic infection or malignancy) induces a state of altered immune profile, transcriptional and epigenetic states, as well as impaired T-cell function. Since a subset of PIRDs amplify T-cell receptor (TCR) signaling and/or inflammatory cytokine signaling cascades, it is possible that they could induce T-cell exhaustion by genetically mimicking chronic infection. Here, we review the fundamentals of T-cell exhaustion and its possible role in IEIs in which genetic mutations mimic prolonged or amplified T-cell receptor and/or cytokine signaling. Given the potential insight from the many forms of PIRDs in understanding T-cell function and the challenges in obtaining primary cells from these rare disorders, we also discuss advances in CRISPR-Cas9 genome-editing technologies and potential applications to edit healthy donor T cells that could facilitate further study of mechanisms of immune dysfunctions in PIRDs. Editing T cells to match PIRD patient genetic variants will allow investigations into the mechanisms underpinning states of dysregulated T-cell function, including T-cell exhaustion.

## Introduction

Inborn errors of immunity (IEIs) are a group of heterogeneous monogenic diseases that disrupt immune function (1–3). The clinical features of IEIs are diverse and include recurrent infections and immune dysregulation, which can present as autoinflammation, autoimmunity, lymphoproliferation, cancer, and/or atopy (1, 4, 5). Flow cytometry and clinical genetic testing (e.g., single gene sequencing, gene panels, whole exome, and genome sequencing) have emerged as powerful tools that aid in the identification and diagnosis of monogenic IEIs (6–9). However, there is significant complexity in the diagnosis of IEIs *via* clinical genetic testing. While genetic testing may yield known pathogenic variants in genes known to cause IEIs, many more patients may have negative results, or testing may uncover variants of unknown significance (VUS), requiring further evaluation for interpretation. In the latter case, complexity comes from the fact that many variants in any given gene may not have any direct impact on protein function, and those that do impact protein function can amplify or reduce a protein’s presence and/or function (referred to as a gain of function (GOF) or loss of function (LOF), respectively) (2, 3, 10). The clinical phenotypes (in GOF vs. LOF) may be more similar than one would expect, so clinical phenotype may not be sufficient for a determination. To understand whether a novel genetic variant in a patient is causative, pathogenic variant requires a context of deeper study of key, interconnected signaling pathways at baseline in healthy controls and comparison to alterations in IEI patients. This context allows clarity when focusing on the impact of a specific genetic variant in an individual patient, from a clinical perspective. More broadly for the field, by studying samples from IEI patients with pathogenic variants in key signaling molecules, we can learn both about those rare resulting IEIs (and help us diagnose novel IEIs) and about how the fundamental pathways of the immune system can be mistuned, either overly amplified or inappropriately muted. Therefore, from the perspective of human immunology research, the opportunity to study these rare patients allows us to work toward both optimizing diagnosis and targeted therapeutics and gaining a deeper understanding of how the impacted immune pathways interact.

**Figure 1:**
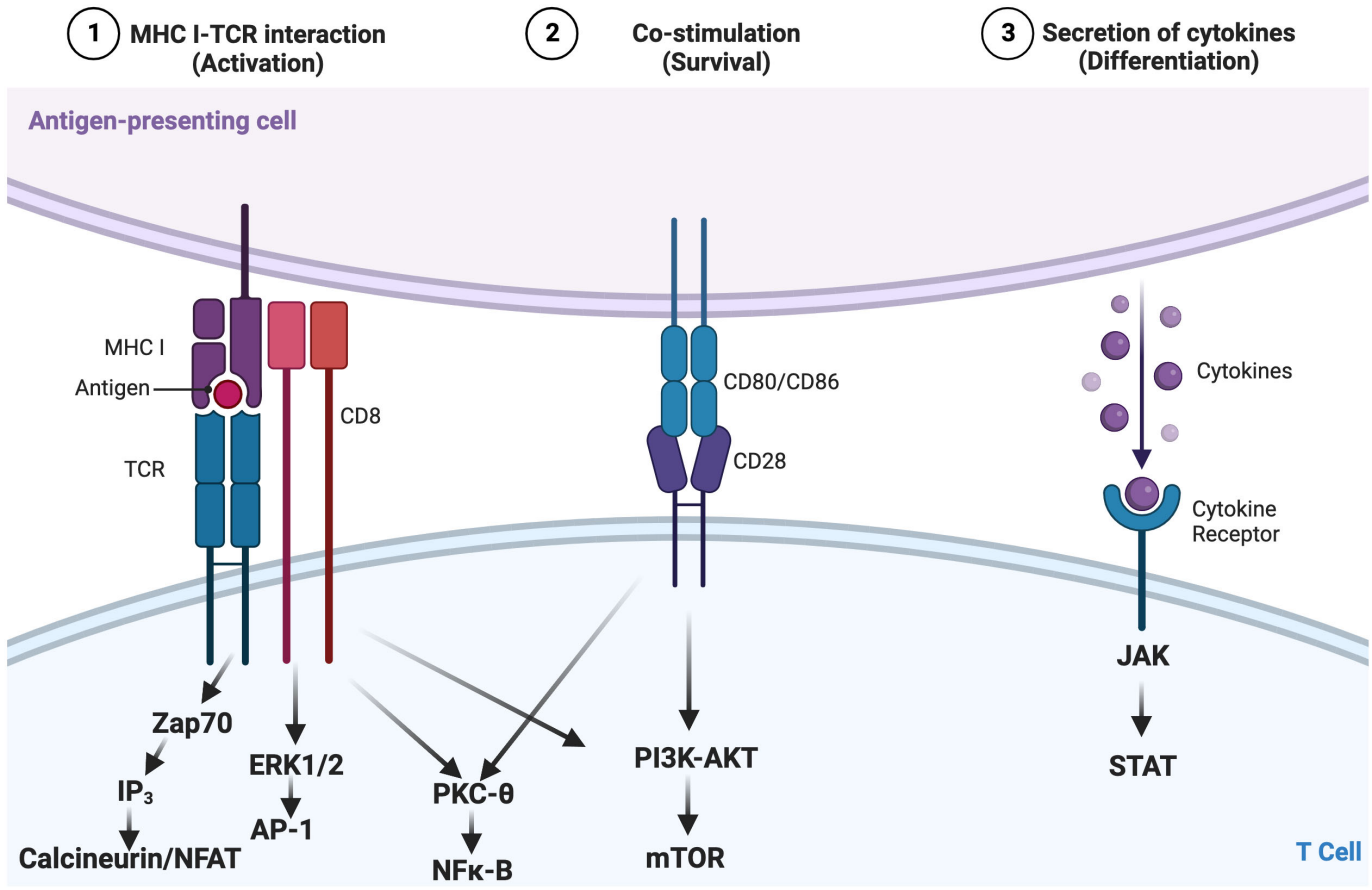
Canonical three-signal activation model for T cells. Activation is dependent on integration of three signals: 1) recognition by the T-cell receptor (TCR) of an antigen presented as a peptide by the major histocompatibility complex (pMHC) on antigen-presenting cells (APCs); 2) costimulation, which lowers the stimulation threshold of naïve T cells, prevents anergy, and enhances cytokine production; and 3) cytokine signaling along the JAK-STAT pathway, which amplifies the clonal expansion, survival, and differentiation of T cells. These converging cellular signaling cascades influence the activation and differentiation status of T lymphocytes, in this case, CD8+ T cells. Adapted from “Three Signals Required for T cell Activation”, by BioRender.com (2022). Retrieved from https://app.biorender.com/biorender-templates/t-5f7c7b883b9a9000abcf98e4-three-signals-required-for-t-cell-activation.

T cells are a key component of the cellular adaptive immune system and contribute to responses against pathogens and tumors (11). How T cells become activated, differentiate, and ultimately exert their effector functions is governed by various signaling pathways. These pathways include activation *via* the T-cell receptor (TCR; signal 1; **Figure 1**) binding of cell surface costimulatory receptors to their ligands, providing additional signals required to enhance the magnitude of T-cell activation and prevent anergy (signal 2; **Figure 1**), and soluble mediators, including cytokines (signal 3; **Figure 1**), which impact the differentiation, efficacy, and durability of the T-cell response (**Figure 1**) (12–15). Regulated T-cell responses ensure effective immunity while preserving immune tolerance (16–18). Altered TCR signaling may impact primary responses to infection and malignancy or foster autoimmunity by hyperactivating autoreactive T cells and impairing mechanisms of tolerance [e.g., central tolerance, anergy, activation-induced cell death, and regulatory T cells (Tregs)] (13, 19–23). Therefore, changes in signaling can lead to altered states of activity and cellular fates and even impact the survival of the host.

Within IEIs, primary immune regulatory disorders (PIRDs) alter immune signaling pathways and result in immune dysregulation (5, 24). Given the role that T cells play in establishing and maintaining immune responses, homeostasis, and memory, it is important to study the factors and changes that impair T-cell function. There are many impacts on both CD4^+^ and CD8^+^ T cells in PIRDs, but we are fascinated by the shared manifestation in many PIRDs of increased respiratory viral infections and herpes family viral infections. Therefore, here, we will focus on CD8^+^ T-cell dysfunction, specifically T-cell exhaustion, which could contribute to susceptibility to these types of infections in IEIs (25–28). We will review the fundamentals of T-cell exhaustion, evidence of exhaustion in IEIs, and strategies that will allow a more complete evaluation of the role of T-cell exhaustion in IEIs.

## Relationship between inborn errors of immunity and T-cell exhaustion

### What is T-cell exhaustion?

**Figure 2:**
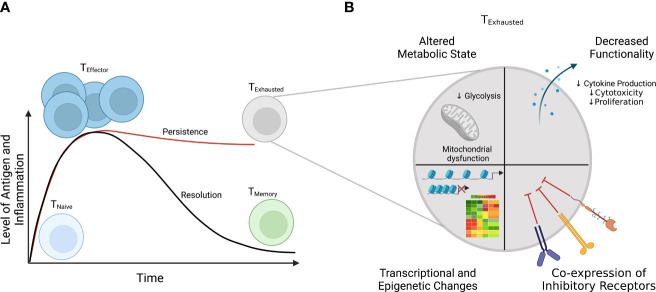
Differential T-cell responses to short-term (acute) and long-term (chronic) infections. **(A)** In acute infections (solid black line), T-cell response spikes are marked by acquisition of effector function (e.g., cytokine production) and upregulation of activation markers and clonal expansion. When an infection or antigen is cleared, a small subset of effector T cells differentiate into memory cells, poised to reactivate upon re-infection, while most cells die. However, if the antigen level or inflammation persists as in the case during chronic infections, T cells develop into a distinct subset, termed “exhausted” T cells (solid red line). **(B)** Among the characteristics that define exhausted T cells are 1) coordinate expression of multiple immunoregulatory receptors, 2) decreased effector function, 3) altered metabolic function, and 4) altered transcriptional and epigenetic status.

Upon encountering antigen presented by antigen-presenting cells and inflammatory cues (e.g., cytokines), naïve CD8^+^ T cells are rewired to activate, rapidly proliferate, and become effectors (**Figure 2A**) (29). When the pathogen or antigen is cleared, a minority of these effector cells survive and become memory cells, while the vast majority die *via* apoptosis (29). However, when antigen and inflammation persist, as in the setting of chronic viral infection or malignancy, T cells can become exhausted (**Figure 2**) (25–27). The amount or the length of exposure to these signals has been shown to connect to the extent of T-cell exhaustion (30). Indeed, in some settings, cessation of signaling enables cells to reverse/reduce the extent of exhaustion and regain some aspects of functionality (30, 31). Though antigen stimulation is widely recognized as a driver of exhaustion, inflammatory signals (e.g., cytokines) and non-immune signals, such as hypoxia, also can contribute to the development of exhaustion (32, 33). Among its many characteristics, T-cell exhaustion involves an altered immune phenotype, including increased and sustained expression of multiple inhibitory receptors, which can include increased levels of PD-1, CTLA-4, LAG-3, and TIM-3 (27, 34). While alterations in immune phenotype can imply a functional impact, these changes are not sufficient to define T-cell exhaustion, as some of these makers are also expressed during the course of normal T-cell activation; therefore, functional changes must also be evaluated. Functional assays are central to testing suspected T-cell exhaustion, including the loss of effector cytokines such as IL-2, TNF-α, and IFN-γ; impaired cytotoxicity; and decreased proliferation (35, 36). Additionally, T-cell exhaustion is characterized by dysregulated transcriptional and epigenetic circuitry (e.g., increased expression of TOX) and alterations in metabolic state, including decreased mitochondrial function and defects in glycolysis (**Figure 2B**) (32, 37–45).

However, T-cell exhaustion is by no means the only state of T-cell dysfunction. T-cell senescence, often mentioned as a comparator to T-cell exhaustion, is an entirely separate process. The definitions of senescence and exhaustion can seem unclear in the literature, because both states share several phenotypic and functional characteristics, though they are fundamentally different (46). Hallmarks of senescent T cells include changes in phenotype (e.g., upregulation of CD45RA, CD57, and KLRG1 and absence of CD28), shortened telomeres, and cell cycle arrest (46–50). While exhausted and senescent CD8^+^ T cells have defects in proliferative potential, senescent CD8^+^ T cells still retain some effector functions, including the ability to produce high levels of both pro- and anti-inflammatory cytokines compared to hypofunctional exhausted CD8^+^ T cells (46, 51, 52). In addition, senescence is not currently understood to be recoverable. Senescence has been observed in various IEIs, including activated phosphoinositide 3-kinase δ syndrome (APDS), chronic granulomatous disease (CGD), and tripeptidyl peptidase II deficiency (53–57). While understanding the underlying mechanisms that lead to the development of dysfunctional T-cell states such as exhaustion and senescence are important, here we will focus on T-cell exhaustion, in part due to the potential for recovering some effector functions in this cell state.

### Aberrant signaling networks in primary immune regulatory disorders

While each IEI is a unique genetic disease, variants in different genes can converge in multiple IEIs with similar impacts on shared signaling pathways. This could potentially yield shared dysfunctional states and similar clinical phenotypes (58). For example, within IEIs, this could be anticipated to take place in PIRDs that amplify TCR signaling and/or inflammatory cytokine signaling. As previously stated, multiple coordinated signals are required to activate a T cell and control its function (**Figure 1**) (12). Therefore, certain PIRDs that amplify signaling downstream of the TCR can thus cause T-cell hyperactivation. We, as well as others, have found that this can lead to exhaustion-like phenotypes (49, 53, 59, 60). For example, assessment of a cohort of common variable immunodeficiency (CVID) patients, including some with monogenic PIRDs, through flow and mass cytometry has revealed hallmarks of CD8^+^ T-cell exhaustion including co-expression of inhibitory receptors, loss of markers such as CD127 and CD28, and expression of transcriptional and epigenetic regulators such as TOX (61).

Now, we will focus on how T-cell hyperactivation in PIRDs could theoretically happen in two broad ways—blocking negative signals or amplifying positive signals.

First, there could be a decrease in inhibitory signaling; here we will review some examples. The first three examples are closely related: autosomal dominant loss of function mutations in *CTLA4* (also known as CTLA-4 haploinsufficiency; CTLA-4^+/−^), LRBA deficiency, and DEF6 deficiency (62–65). CTLA-4 is expressed by T cells (e.g., Tregs and activated cells) and competes with CD28 for ligands, CD80/CD86, which are expressed on antigen-presenting cells (APCs) (66–69). In addition, CTLA-4 can remove and degrade CD80/CD86 from the surface of APCs through a process called trans-endocytosis, blocking costimulation to T cells (70). The balance of positive and negative signals exerted by CD28 and CTLA-4 is crucial for regulating T-cell responses (66, 67, 71). Immunologic features of CTLA-4^+/−^ include, but are not limited to, a reduction in the number of CD4^+^ T cells, alterations in the expression of inhibitory receptors and activation markers on CD8^+^ T cells (e.g., upregulated expression of PD-1 and HLA-DR), alterations in the B-cell compartment (e.g., decreased counts of total B cells and switched memory B cells and increased frequency of CD21^low^ B cells), as well as increased apoptosis of both T and B cells *in vitro* (60, 72, 73). *LRBA* plays a role in the recycling of CTLA-4 and LRBA deficiency results in a reduction of CTLA-4 levels. DEF6 deficiency affects CTLA-4 cycling dynamics and uptake of CD80, clinically and functionally resembling CTLA-4 haploinsufficiency and LRBA deficiency (62, 64). Similarly related, as PD-1 can act as a negative regulator of T-cell function, loss of PD-1 could result in hyperactive cells. A patient with homozygous LOF in *PDCD1* was recently reported with autoimmune manifestations and tuberculosis (TB) (74). Interestingly, while this patient expressed hallmarks consistent with hyperactivation, such as HLA-DR and CD38, the patient’s cells did not produce IFN-γ upon stimulation, including within the CD8^+^ T-cell population (74). Finally, another example of a monogenic disorder that results in the loss of a negative regulator of T-cell receptor signaling involves the ubiquitination of T-cell receptors and proximal signaling molecules such as TCRζ and MEKK1 (75–77). Mutations in *ITCH*, which encodes itchy E3 ubiquitin protein ligase, have been described; these patients present with immune dysregulation including multisystem autoimmune disease and immune deficiency (4, 78–81). Thus, each of these IEIs that reduce inhibitory signaling could theoretically cause chronic antigen-like signaling and could thus potentially cause T-cell exhaustion.

Second, there can be increased overall activating signals; here we will review a few examples. Clinically, APDS often presents as a monogenic form of CVID, with a variable phenotype that includes recurrent infections, autoimmunity, lymphadenopathy, and chronic viral infections (e.g., Epstein–Barr virus (EBV) and cytomegalovirus (CMV)) (82–84). APDS is caused by heterozygous mutations in *PIK3CD* (GOF) or *PIK3R1* (LOF); in both cases, there is an amplification of PI3K signaling (49, 53, 85). While *PIK3R1* encodes regulatory subunits of PI3K and is more ubiquitously expressed, *PIK3CD* encodes p110δ and is more restricted to the hematopoietic lineage (86, 87). Signal transduction along the PI3K pathway regulates various aspects of immune cell homeostasis and activity, including the ability of a cell to initiate changes in its metabolism, grow and differentiate, and function. Conversion of the molecule phosphatidylinositol 4,5-biphosphate (PIP2) to phosphatidylinositol 3,4,5-triphosphate (PIP3) through phosphorylation by PI3Kδ leads to AKT recruitment and mTOR pathway activation (82, 88). This pathway is negatively regulated by PTEN, which converts PIP3 to PIP2; APDS-like immune dysregulation has been observed in patients with PTEN LOF variants (83, 88–90). Thus, mutations in *PIK3CD*, *PIK3R1*, and *PTEN* result in hyperactivation of the PI3K/AKT/mTOR pathway, causing amplification of downstream members of the TCR signaling cascade.

Another way of altering activating signals is through amplified inflammatory cytokine signaling through the Janus kinase-signal transducer and activator of transcription (JAK-STAT) pathway. The JAK-STAT pathway and inflammation regulate various aspects of the immune system, including the polarization and function of T cells (91–93). Genetic alterations that lead to persistent or prolonged cytokine signaling such as GOF mutations in STAT1 and STAT3 (STAT1 GOF and STAT3 GOF) have been described in patients with immune dysregulation (94–97). The clinical phenotype of STAT1 GOF is variable and includes autoimmunity, autoinflammation, and increased susceptibility to both chronic mucocutaneous candidiasis (CMC) and viral infections (94, 95). Meanwhile, STAT3 GOF leads to a complex immune dysregulatory picture with multi-organ autoimmunity, lymphoproliferation, and recurrent infections (94, 96–100). Whether genetically amplified or environmentally inflammatory cytokine signaling in human T cells is sufficient to lead to T-cell exhaustion remains unknown.

### What is the evidence for T-cell exhaustion in inborn errors of immunity?

While exhaustion has been studied in the context of chronic viral infection and malignancy, evidence consistent with exhaustion has been found in various IEIs (**Table 1**), ranging from those altering PI3K and NF-κB signaling to other key molecules involved in T-cell activation and inhibition. In APDS, there is an elevated expression of PD-1 on CD8^+^ T cells (59). Because T-cell exhaustion cannot be defined by one marker alone, expression of additional inhibitory molecules, including CD160 and CD244, has also been interrogated; these markers have been shown to be increased in patients (56, 59). Increased susceptibility to apoptosis, which is associated with T-cell exhaustion, has also been observed in APDS (57, 82, 101, 102, 107, 108). In one study, decreased cytotoxicity of EBV-specific CD8^+^ T-cell lines against lymphoblastoid cell lines (LCLs) was observed (57). In another study, functional testing of CD8^+^ T cells from APDS patients demonstrated impaired function (i.e., reduction in IL-2 secretion and proliferation), which improved with rapamycin treatment (53). *In vitro*, blocking the PD-1/PD-L1 pathway increased the function (proliferation and cytokine production) of EBV-specific CD8^+^ T cells (59). This suggests the possibility of T-cell exhaustion in APDS, as immune checkpoint blockade improved some aspects of function. Though not the focus of this review, there is also evidence of B-cell immunometabolic dysregulation, with block in progression from transitional to follicular B-cell stage of differentiation secondary to metabolic alterations in APDS (109). These data raise the question of how GOF mutations affecting PI3K/AKT/mTOR signaling might affect the various stages of T-cell activation and differentiation and whether this may cause CD8^+^ T-cell exhaustion.

**Table 1:** Summary table of data suggesting T-cell exhaustion in various IEIs.

Condition	Evaluation of exhaustion	References
**APDS**	Immune phenotype: ↑ PD-1, CD160, CD244 Function: ↓ cytotoxicity, IL-2, proliferation Other: ↑ apoptosis Rapamycin treatment: ↑ IL-2, proliferation PD-L1 blockade: ↑ cytokine, proliferation	Lucas et al. (2014) (53) Cannons et al. (2018) (56) Edwards et al. (2019) (57) Wentink et al. (2018) (59) Angulo et al. (2013) (101) Wentink et al. (2017) (102)
**Trisomy 21**	Immune phenotype: ↑ PD-1, CD160, CD244	Peeters et al. (2022) (103)
***CARD11* GOF**	Function: ↓ proliferation	Snow et al. (2012) (104)
**DOCK8 deficiency**	Immune phenotype: ↑ PD-1, CD244 Function: ↓ proliferation, cytokine (IL-2, TNF-α, and IFN-γ), cytotoxic molecules (CD107a, Granzyme A, and Granzyme B)	Pillay et al. (2019) (105) Randall et al. (2011) (106)
***PDCD1* deficiency**	Immune phenotype: ↑ TIGIT Function: ↓ cytokine (IFN-γ, TNF-α)	Ogishi et al. (2021) (74)
**CVID**	Immune phenotype: ↑ PD-1, TIGIT, CD244, LAG-3 Transcriptional and epigenetic factors: ↑ TOX, eomes Function: ± cytotoxic and proinflammatory molecules	Klocperk et al. (2022) (61)

APDS, activated phosphoinositide 3-kinase δ syndrome; CVID, common variable immunodeficiency; IEIs, inborn errors of immunity.

Upward arrow (↑) means upregulated/increased; Downward arrow (↓) means downregulate/decreased.

In other IEIs, there may be evidence of exhaustion, though this remains to be fully tested, and exact mechanisms have yet to be elucidated. In trisomy 21, while a surface immune phenotype consistent with exhaustion has been identified (e.g., increased PD-1, CD244, and CD160), a functional impact remains to be measured (103). Another pathway in which dysregulation could potentially lead to an exhausted-like picture is the NF-κB family of transcription factors, which regulates various processes, including immune function and inflammatory responses, cell growth, and apoptosis (110). Heterozygous GOF variants in *CARD11* result in BENTA (B-cell expansion with NF-kB and T-cell anergy), which increases activity in NF-κB and yields impaired T-cell proliferative responses following CD3/CD28 stimulation, perhaps reflective of an exhausted-like state, though parameters that define exhaustion, such as increased expression of inhibitory receptors, remain to be tested (104, 111). Exhausted-like phenotypes have been observed in DOCK8-deficient patients, including decreased proliferation and cytokine secretion, and increased expression of the molecules PD-1 and 2B4 (105, 106). Decreased IFN-γ production (and trending decrease in TNF-α) has been described in a patient with *PDCD1* deficiency; in CD8^+^ T cells, there was a slight elevation in TIGIT expression within the CD8^+^ T_EM_ and T_EMRA_ populations (74). Therefore, with possible evidence of an exhausted-like phenotype in various IEIs (**Table 1**), we must move forward mechanistically to test whether exhaustion-like outcomes are caused by the genetic variant itself in a cell-intrinsic fashion or the result of cell-extrinsic physiological insults including recurrent infections and/or autoimmunity or if both scenarios contribute to this phenotype.

## Using CRISPR-Cas9 editing to facilitate further studies of T-cell exhaustion in inborn errors of immunity

With all that remains to be learned, it is necessary to devise strategies to allow the deep study of IEIs caused by these signaling pathways in human cells and test key hypotheses. Given the challenges in obtaining sufficient primary T cells from these rare IEI patients to deeply delve into mechanistic questions, especially from untreated patients, can we develop tools that will result in stable genetic modification of primary cells, or do we need to rely on cell lines and model organisms? We will next consider how gene editing technologies can be leveraged to edit healthy donor T cells to match patient genetic variants to provide the opportunity to study the impact of acute and chronic alterations in these immune pathways, as well as the impacts of modulating those pathways on cell function *in vitro*.

Gene therapy is a precision approach to treating IEIs. In a subset of IEI disorders including adenosine deaminase deficiency severe combined immune deficiency (ADA-SCID), X-linked SCID (X-SCID), CGD, and Wiskott–Aldrich syndrome, autologous hematopoietic stem cell transplantation (HSCT) in combination with gene therapy has been evaluated in clinical trials and found to be an effective therapeutic strategy (112–119) Historically, gene therapy involved the introduction of a corrected gene into affected cells, but integration happened randomly and not necessarily at its native site. However, gene editing tools that allow for site-specific editing have recently and rapidly emerged and been developed to overcome the limitations and risks of conventional gene therapy, such as integration at random sites in the genome, which could result in malignancy (120, 121).

**Figure 3:**
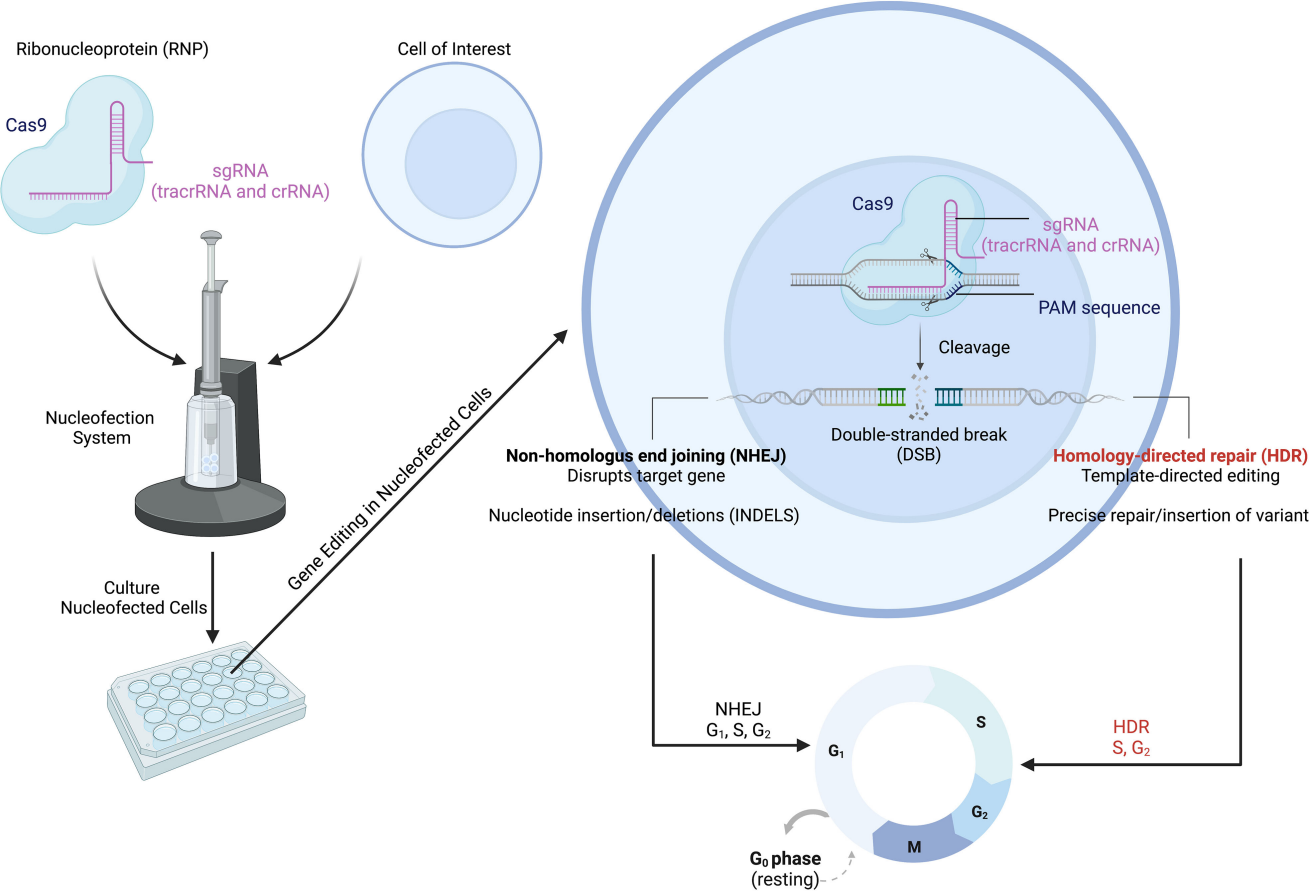
CRISPR-Cas9 gene editing to model IEIs/PIRDs. The CRISPR-Cas9 system consists of the Cas9 protein in complex with a targeting gRNA, forming a ribonucleoprotein (RNP). Specificity is conferred through various mechanisms including the sgRNA and presence of the PAM sequence. The Cas9 opens and cuts the targeted sequence on both strands to generate double-strand breaks (DSBs). This activates DNA repair pathways (NHEJ and HDR) in cells and results in modifications at a target locus. The NHEJ pathway can be used for scenarios in which genetic variants result in the loss of expression of an encoded protein, while HDR can be employed for introduction of missense mutations. IEIs, inborn errors of immunity; PIRDs, primary immune regulatory disorders; sgRNA, single-guide RNA; PAM, protospacer adjacent motif; NHEJ, non-homologous end joining; HDR, homology-directed repair. Adapted from “CRISPR/Cas9 Gene Editing”, by BioRender.com (2022). Retrieved from https://app.biorender.com/biorender-templates/t-5f873df466346900a43c6db1-crisprcas9-gene-editing.

Of the various genome editing tools, CRISPR-Cas9 is one of the most versatile gene editing platforms with the best currently available precision and efficiency (121–123). The CRISPR-Cas9 system involves two main components that make up a ribonucleoprotein (RNP) complex that allows for gene editing: the Cas9 endonuclease and a single-guide RNA (sgRNA) (**Figure 3**) (124). Within the sgRNA are the CRISPR RNA (crRNA) and trans-activating RNA (tracrRNA) (125–127). The tracrRNA allows Cas9 to bind and form the RNP, while the crRNA targets the endonuclease to a specific location in the genome (**Figure 3**) (124). Additional specificity is provided by the protospacer adjacent motif (also called the PAM sequence), which needs to be present in order for the Cas nuclease to cut (124). The Cas endonuclease introduces a double-strand break (DSB) at the target site; this DSB then activates DNA repair pathways (126, 128, 129). The DSB can be repaired by one of two mechanisms: non-homologous end joining (NHEJ) or homology-directed repair (HDR). NHEJ is imprecise, leading to the insertion or deletion of nucleotides (i.e., INDELs), which disrupt the gene of interest, while HDR results in nucleotide changes at precise locations in the genome (129–132). For HDR to occur, a donor template containing the new, modified DNA sequence needs to be provided; the sequences around this site on the donor template must be homologous to the target DNA sequence (129–132). While NHEJ can occur during any phase of the cell cycle, HDR only happens during the G2/S cell cycle phases (**Figure 3**) (128). The HDR pathway allows a wide range of editing: from single nucleotides to the insertion of whole cDNAs of varied lengths (122, 133). Although here we have focused on DNA cleavage-induced editing, alternative techniques using CRISPR-Cas technology exist such as base editing (directly alters the chemical sequence of the DNA without DSBs), prime editing (generates RNA template for gene alteration), and CRISPR interference and activation (for transcriptional control) (134–138). However, many limitations have impeded the full deployment and use of CRISPR-Cas9, especially when aiming to utilize the HDR pathway; we will next discuss some of these limitations.

To repair an IEI-causing gene variant, various variables need to be considered and optimized. These include a selection of the proper editing system, timing of when editing reagents are introduced, and the quantity of reagents to yield a high degree of editing in cells of interest, while ideally having negligible cytotoxicity and off-target effects. To address this, different strategies have been tested to circumvent some limitations of HDR-based genome-editing strategies, such as its low editing efficiency. For example, as NHEJ is the dominant repair mechanism in cells, small molecules (e.g., those that synchronize cells at the S/G2 stage) or fusion proteins that promote HDR and/or inhibit NHEJ have been developed and used to promote HDR (139–148). Recently, CRISPR-Cas9 editing was used in human hematopoietic stem and progenitor cells (HSPCs) to correct Wiskott–Aldrich syndrome *via* the HDR pathway (149). These data suggest that targeted gene editing is achievable and can be leveraged in the study and correction of IEIs.

Nevertheless, while gene editing has been used to correct alterations in genes to directly treat IEIs, here we propose introducing relevant patient mutations into healthy donor primary cells of interest to perturb immune signaling pathways to match IEI patient cells (138, 150). This strategy would allow researchers to investigate how these variants dysregulate immune cells and their functions, especially prior to immune-relevant therapy, and increase the number of cells available for mechanistic studies and investigations into the impact of perturbations, especially for rare patients or patients with lymphocytopenia.

As previously discussed, persistent antigen and/or inflammatory signals have been implicated in T-cell exhaustion. However, whether the presence of genetic mimics of chronic activation in T-cell signaling pathways is sufficient to yield T-cell exhaustion has not been proven—IEIs provide novel strategies for assessing the mechanisms of altered cell signaling that can yield this T-cell state. Specifically, missense variants that activate T-cell signaling pathways could be introduced into primary CD8^+^ T cells using CRISPR-Cas9 mechanisms coupled with repair templates (e.g., double-stranded DNAs (dsDNA) or single-stranded donor oligonucleotides (ssODNs)) (85, 122, 151). However, a balance between increasing the frequency of HDR while only modifying a single allele (as a multitude of IEI are heterozygous) must be achieved. Changing the distance between the cut site and desired mutation location (referred to as “cut-to-mutation distance”) has been shown to affect zygosity (152). In practice, to confirm the successful introduction of a variant (e.g., GOF as an example), sequencing plus an appropriate readout (e.g., phospho-flow cytometry) can be employed. Once the cells are shown to match patient cells genetically, altered immune profile receptors (e.g., upregulated PD-1) and any alterations in T-cell function, including loss of effector function (cytokine production and proliferation), can be assessed (59). For a genetic variant leading to the loss of an encoded protein (and thus absent or decreased expression), the gene of interest can be knocked out to reveal its function in cells of interest such as primary CD8^+^ T cells and confirmed by flow cytometry or Western blotting and differences in phenotype and function assessed (73). However, it is worth noting that acute changes in signaling may not be sufficient to fully recapitulate the alterations observed in primary patient cells. Therefore, the use of prolonged *in vitro* cultures, restimulations, and comparison to unedited cells (e.g., cells that received a non-targeting control guide) may be needed to fully dissect the impact of these variants on immune cell phenotype and function.

**Figure 4:**
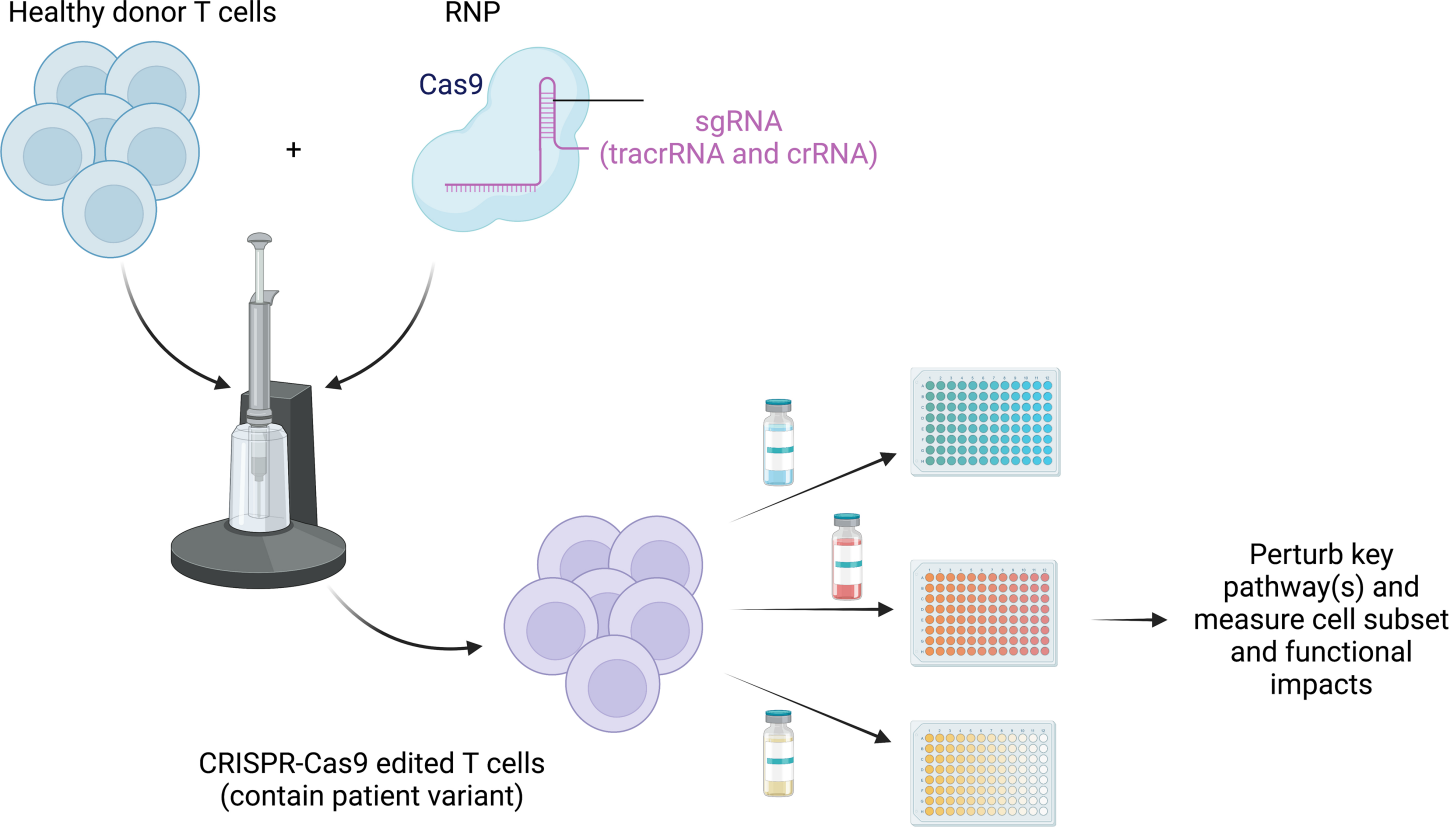
Retuning altered pathways to improve function. CRISPR-Cas9 can be used to edit healthy donor primary T cells to match the patient variant to overcome paucity of patient cells, especially those prior to immune-relevant therapy. Testing of treatment options (both preexisting and novel) can be exploited using high-throughput screening strategies to study the impact of blocking and/or stimulating identified dysfunctional pathways. This will allow us to identify and target common states of dysfunction across monogenic diseases.

By harnessing the power of CRISPR-Cas9 gene editing, scientists can directly perturb core signaling pathways in healthy control T cells, matching the disease-causing variants in human genes found in IEIs, to provide additional human cells for mechanistic testing and therapeutic target testing. With the use of this resource, these dysregulated pathways underlying impaired function can theoretically be “retuned” *in vitro* by blocking and/or amplifying the affected pathways and assessing for improvements in baseline functional deficits (**Figure 4**). This could be achieved by targeting the altered protein itself, a component of the signaling pathway upstream or downstream, or a related pathway that could be affected by the altered signaling in each IEI. As discussed above in APDS, Wentink et al. demonstrated that blocking the PD-1/PD-L1 signaling pathway improved function in antigen-specific CD8^+^ T cells, while Lucas et al. used rapamycin to reduce PI3K signaling and improve T-cell function (53, 59). Evidence of “retuning” signaling pathways in IEI has also been described in patients with STAT3 LOF or STAT1 GOF mutations; both conditions share impaired differentiation of Th17 cells, which has been shown to be a result of upregulation of PD-L1 (153–156). Blocking PD-L1 was shown to partially restore both the levels of IL-17A protein and transcript in these patients (156). Together, these data demonstrate that the (dys)functional state of immune cells can be manipulated in PIRDs. However, deepening these types of investigations is very challenging given how rare these diseases are and how challenging it can be to study cells from untreated patients. Therefore, to better understand these key biological and medical questions around T-cell exhaustion in IEI, and to take an expanded approach to test possible novel therapeutics *in vitro*, the strategy of creating edited healthy control T cells that match patient variants may be transformative.

## Conclusion

Primary immune regulatory disorders provide unique insights into the impacts of alterations in individual signaling molecules in T cells on their function. In a subset of PIRDs, there is evidence of T-cell exhaustion, but the contribution of specific altered pathways to the induction or maintenance of cellular fates, such as exhaustion, is not fully understood. Given the challenges in obtaining primary cells from these rare disorders, genome-editing strategies such as CRISPR-Cas9 can be leveraged to edit healthy donor T cells to contain patient genetic variants found in various PIRDs, providing additional opportunities to study these rare disorders and perform mechanistic studies. Identifying the targetable cellular fates induced by causative gene variants in IEI and potentially malleable, relevant pathways (e.g., PD-1 blockade) in IEI will allow us to evaluate the impact and best strategies for retuning pathways in IEI patients in conjunction with high-throughput screening strategies, providing potential novel therapeutic agents. These perturbation studies aimed at restoring function can be optimized in CRISPR-Cas9 edited cells and then confirmed in primary cells from patients. CRISPR-Cas9 gene editing could thus expedite our understanding of signaling pathways and mechanisms of T-cell dysfunction in IEIs. Defining shared dysfunctional states across rare IEIs (or other rare diseases), using gene editing in healthy control human cells to match patient variants, and identifying strategies to retune dysfunctional states may lead to improvements in patient treatment. Additionally, these strategies may increase our understanding of basic immunology and immune dysregulation processes in these rare genetic diseases and other more common diseases, including autoimmunity, chronic infection, and chronic inflammatory disorders.

## Author contributions

SEH and JSC planned the manuscript together, JSC drafted it and SEH and JSC both edited it. All figures created with BioRender.com. All authors contributed to the article and approved the submitted version.

## Funding

JSC: University of Pennsylvania Presidential Fellow, HHMI Gilliam Fellow; SEH: National Institute of Allergy and Infectious Diseases (NIAID) K08AI135091, Burroughs Wellcome Fund CAMS. Faculty Development Award.

## Conflict of interest

The authors declare that the research was conducted in the absence of any commercial or financial relationships that could be construed as a potential conflict of interest. All figures created with BioRender.com.

## Publisher’s note

All claims expressed in this article are solely those of the authors and do not necessarily represent those of their affiliated organizations, or those of the publisher, the editors and the reviewers. Any product that may be evaluated in this article, or claim that may be made by its manufacturer, is not guaranteed or endorsed by the publisher.
